# Solute Diffusivity and Local Free Volume in Cross-Linked Polymer Network: Implication of Optimizing the Conductivity of Polymer Electrolyte

**DOI:** 10.3390/polym14102061

**Published:** 2022-05-18

**Authors:** Yi-Chen Tsai, Chi-Cheng Chiu

**Affiliations:** 1 Department of Chemical Engineering, National Cheng Kung University, Tainan 701, Taiwan; n38091508@gs.ncku.edu.tw; 2 Hierarchical Green-Energy Materials (Hi-GEM) Research Center, National Cheng Kung University, Tainan 701, Taiwan; 3 Fire Protection and Safety Research Center, National Cheng Kung University, Tainan 711, Taiwan

**Keywords:** solute diffusion, cross-linked polymers, Vogel–Tammann–Fulcher equation, free volume theory, molecular dynamics

## Abstract

The diffusion of small molecules or ions within polymeric materials is critical for their applications, such as polymer electrolytes. Cross-linking has been one of the common strategies to modulate solute diffusivity and a polymer’s mechanical properties. However, various studies have shown different effects of cross-linking on altering the solute transports. Here, we utilized coarse-grained molecular dynamics simulation to systematically analyze the effects of cross-linking and polymer rigidity of solute diffusive behaviors. Above the glass transition temperature $T_{g}$, the solute diffusion followed the Vogel–Tammann–Fulcher (VTF) equation, D = D${}_{0}$ e${}^{-E_{a}/R\left(T-T_{0}\right)}$. Other than the conventional compensation relation between the activation energy $E_{a}$ and the pre-exponential factor D${}_{0}$, we also identified a correlation between $E_{a}$ and Vogel temperature T${}_{0}$. We further characterized an empirical relation between T${}_{0}$ and cross-linking density. Integrating the newly identified correlations among the VTF parameters, we formulated a relation between solute diffusion and the cross-linking density. The combined results proposed the criteria for the optimal solute diffusivity in cross-linked polymers, providing generic guidance for novel polymer electrolyte design.

## 1. Introduction

Currently, polymers have been widely applied in various fields, such as coating materials, membranes and filters, storage devices, optical films, and electronic devices, etc. When apply as a protective coating and filtering membrane, the diffusion of small molecules or ions within polymers is one of the critical properties [1,2,3]. Many recent studies in high-power and high-safety lithium batteries have utilized functional polymers to fabricate novel solid polymer electrolytes (SPEs), in which the ion conductivity is also the key property [4,5]. Therefore, modulating polymers to optimize the diffusivities and/or selectivities of solvated molecules/ions has been one of the main research focuses over the past few decades.

Various strategies have been applied to modulate the solute diffusivity within the polymer matrix, including tuning the nature of polymers and solutes, the addition of plasticizers or fillers, and cross-linking polymer networks [1]. The transport properties mainly depend on the free volume of solute within the polymer hosts and the segmental motions of the polymer chains [3,6,7]. In general, polymers with low glass transition temperature (T${}_{g}$) have higher chain mobilities with greater diffusivity [7]. Adding plasticizers or fillers, depending on their compatibility with the polymers, can alter the mobilities of polymer segments and the free volume within polymer matrix, leading to the modulation of solute diffusivity [1]. Cross-linking polymers to form network structures improves the overall mechanical properties. Yet cross-links also restrain the polymer segmental motions and, thus, generally reduce solute diffusion [8,9].

Most polymer matrices contain both amorphous and crystalline regions. The diffusion of solute mainly occurs in the amorphous region and is therefore highly related to polymer segmental motions [7]. The glass transition temperature (T${}_{g}$) of a polymer matrix distinguishes two states of amorphous polymers, i.e., the glassy and rubbery states. Below T${}_{g}$, polymer segments behave similarly to solid glass with only local vibrational motions yet lacking segment translations. Thus, the main transport mechanism of a solute within glassy polymers is the hopping action between vacant sites within polymer hosts and is less related to nearly frozen polymer segments [7,10]. Moreover, the solute diffusivity can be described via the Arrhenius-like formula:

(1) $$\begin{array}{c} D\left(T\right)=D_{0}\;e^{-E_{a}/\mathrm{RT}}, \end{array}$$

where T is the temperature, and R is the ideal gas constant. $E_{a}$ denotes the activation energy of diffusion, and a lower $E_{a}$ corresponds to higher solute diffusivity. Pre-exponential factor $D_{0}$ can be considered as the factor for the probability of solute/polymer configurations for solute hopping and is related to the configurational entropy [11]. There exists a correlation, also known as the compensation effect, between $E_{a}$ and the pre-exponential factor $D_{0}$ [11]:

(2) $$\begin{array}{c} \ln(D_{0})=mE_{a}+n, \end{array}$$

where the slope *m* is usually positive. Therefore, the effect of a larger E${}_{a}$ on reducing solute diffusivity D is compensated by increased D${}_{0}$, according to Equation (2). The explanations for the compensation effect in the Arrhenius formula differs for various applications. For the solute diffusion in polymer, one possible origin is entropy–enthalpy compensation [11,12].

As the temperature rises above T${}_{g}$, the polymers gain distinct segmental motions and become ductile with a rubber-like behavior [7]. The solute diffusivity in rubbery polymers is, thus, affected by the polymer segmental movements [13], and they are generally described by an empirical relation known as the Vogel–Tammann–Fulcher (VTF) equation [14,15]:

(3) $$\begin{array}{c} D\left(T\right)=D_{0}\;e^{-E_{a}/R\left(T-T_{0}\right)}, \end{array}$$

where D${}_{0}$ is the pre-exponential factor, and E${}_{a}$ is the pseudo-activation energy of diffusion related to the polymer segmental motions. T${}_{0}$, as known as the Vogel temperature, denotes the temperature at which polymers have zero-mobility and is generally taken as 50 K below $T_{g}$ for polymer electrolytes [15]. One common interpretation for the VTF-type diffusion in polymer relies on the free volume theory [3,16,17,18], in which (T − T${}_{0}$) originated from the volume expansion with temperature. The Arrhenius and VTF modes of diffusion can be easily distinguished via the Arrhenius plot, i.e., plotting lnD against 1/T. A linear correlation in the Arrhenius plot corresponds to the Arrhenius diffusive behavior. In contrast, a curved relation indicates a VTF mode of diffusion with correlations to polymer segmental motions. Recent studies on polymer electrolytes illustrated a compensation relation in the form of Equation (2) between the pre-exponent D${}_{0}$ and $E_{a}$ for the VTF equation. The compensation parameters, i.e., the *m* and *n* in Equation (2), are influenced by various factors including polymer nature and cross-linking network and salt types, etc., [8,9,19,20].

In general, cross-linking the polymers enhances the mechanical strength but reduces the polymer segmental mobility. Moreover, increasing cross-linking density can lead to reduced solute diffusivity in polymer matrix or ion conductivity in polymer electrolyte [8,21]. However, several studies also demonstrated the existence of the optimal cross-linking density with the maximal solute diffusivity/ion conductivity [9,20,22,23]. To elucidate the effects of cross-linking on solute diffusivity within polymer matrix, we conducted a series of coarse-grained molecular dynamics simulations with various polymer cross-linking densities and polymer rigidities. Our simulation data confirmed the conventional compensation relation between $E_{a}$ and ${\mathrm{lnD}}_{0}$ for VTF types of diffusion above $T_{g}$. Additionally, we found a linear correlation between $T_{0}$ and $1/E_{a}$, which can be further interpreted through free volume theory. T${}_{0}$ was also found to be correlated to 1 over linker length 1/L, i.e., an indicator of the cross-linking density. These allowed us to reformulate the VTF equation and correlate the solute diffusivity with the cross-linking density. The combined results illustrate the criterion for the optimal solute diffusivity, providing insights into the optimization strategies of the conductivity of novel polymer electrolyte systems.

## 2. Methods

### 2.1. Coarse-Grained Molecular Simulations for Cross-Linked Polymer

To simulate a cross-linked polymer matrix, we applied a coarse-grained (CG) bead-spring polymer model, which has been commonly used to study the structural, mechanical, rheological, and dynamic properties of co-polymers, polymer blends, and polymer networks [24,25,26,27,28,29,30]. Specifically, the model has been shown to produce the Rouse and entanglement dynamics of polymers, which is critical for solute diffusion in polymer matrix [24]. The interactions between polymer CG beads (type P) are described via the 12-6 Lennard–Jones (LJ) potential:

(4) $$\begin{array}{c} U^{LJ}\left(r_{ij}\right)=4\varepsilon_{ij}\left[{\left(\frac{\sigma_{ij}}{r_{ij}}\right)}^{12}-{\left(\frac{\sigma_{ij}}{r_{ij}}\right)}^{6}\right], \end{array}$$

where $\sigma_{ij}$, $\varepsilon_{ij}$, and $r_{ij}$ are the effective particle size, interaction energy, and the separation distance between particles *i* and *j*, respectively. The interaction is cut-off at $2.5\;\sigma_{ij}$. The neighbor polymer beads are connected by the finitely extensible nonlinear elastic (FENE) bond [24,31]:

(5) $$\begin{array}{c} U^{bond}\left(r_{ij}\right)=-\frac{1}{2}k_{b}R_{0}^{2}ln\left[1-{\left(\frac{r_{ij}}{R_{0}}\right)}^{2}\right]+4\varepsilon_{ij}\left[{\left(\frac{\sigma_{ij}}{r_{ij}}\right)}^{12}-{\left(\frac{\sigma_{ij}}{r_{ij}}\right)}^{6}\right]+\varepsilon_{ij} \end{array}$$

where $k_{b}=30\;\epsilon /\sigma^{2}$ and $R_{0}=1.5\;\sigma$ (in reduced LJ units of energy ϵ and length σ) denote the bond stiffness and the bond divergence length, respectively. The harmonic angle potential was introduced to polymer chains:

(6) $$\begin{array}{c} U^{angle}\left(\theta \right)=\frac{1}{2}k_{\theta }{\left(\theta -\theta_{0}\right)}^{2} \end{array}$$

where $\theta_{0}=180^{\circ }$ denotes the equilibrium angle. Various bending stiffness $k_{\theta }$ values of 0, 0.2, 1.0, 2.0, 4.0, and 6.0 $\epsilon /{\mathrm{radian}}^{2}$ were applied to modulate the polymer bending rigidity.

Two solute species (types A and C) were introduced to mimic the ions and counterions within solid polymer electrolyte. The interactions between like CG species are also described using LJ potentials; whereas the interactions between unlike CG species are modeled with the Weeks–Chandler–Andersen (WCA) potential [30,32]:

(7) $$\begin{array}{c} U^{WCA}\left(r_{ij}\right)=\left\{\begin{array}{cc} 4\varepsilon_{ij}\left[{\left(\frac{\sigma_{ij}}{r_{ij}}\right)}^{12}-{\left(\frac{\sigma_{ij}}{r_{ij}}\right)}^{6}\right]+\varepsilon_{ij}, & r<r_{c_{ij}}\\ 0, & r\geq r_{c_{ij}} \end{array}\right. \end{array}$$

where the cut-off distance $r_{c_{ij}}$ is 2${}^{1/6}\;\sigma_{ij}$. The attraction between solute A and C was assigned with a higher value of 2 ϵ to account for attractions between counterions. For polymer electrolyte systems, polymers generally have strong interactions with the conducting ions to solvate target salts. Analogously, we increased the interaction strength between polymer P and solute C to simulate the higher affinity between polymer and the conducting solute C. Table 1 lists the complete non-bonded interaction parameters for all CG species.

**Figure 1:**
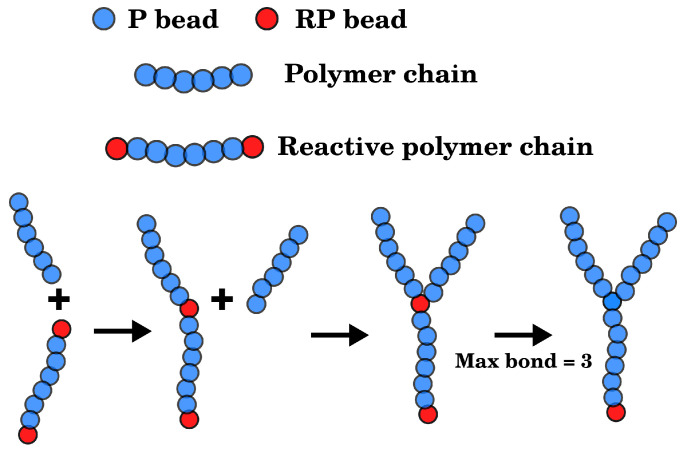
Scheme for the cross-linking reaction between the RP end of a reactive polymer and the P end from a regular polymer. After forming a total of three bonds with P, the RP transforms into P type and loses its reactivity.

To generate the cross-linked network structures, we started with linear polymer chains mixed with two solute species, i.e., C and A. Two types of linear polymers were blended: (1) polymers with only P type CG beads and (2) reactive poly-P polymers of the same length with additional reactive polymer (RP) beads at two ends, where the non-bonded interaction parameters for RP and P were identical. To mimic the in situ cross-linking process such as the photo-induced free radical polymerization [33], a new bond was formed during the molecular dynamics simulation between RP and P beads of different chains when their distance was less than 1.12 σ, i.e., the approximate cut-off distance of the WCA potential. Once a RP terminal formed two additional bonds with P beads from other polymer chains, it was then transformed into type P and lost its reactivity. The scheme for the cross-linking process is illustrated in Figure 1. When fully cross-linked, each RP is linked with three polymer chains. By controlling the ratio of polymers and reactive polymers, we generated polymer networks with different cross-linking densities. In this work, we evaluated Link% as follows:

(8) $$\begin{array}{c} \mathrm{Link}\%=\frac{N_{RP}}{N_{P}+N_{RP}}\times 100\%, \end{array}$$

where $N_{P}$ and $N_{RP}$ denoted the numbers of P and RP beads, respectively. Here, the total numbers of P beads and A-C solute pairs were fixed at 30,000 and 1875, respectively, to control the polymer to solute ratio at 16:1. In a fully linked system, each RP has three bonds and each P has two bonds. Thus, the averaged length for a linker connecting two RP beads can be calculated as follows:

(9) $$\begin{array}{c} L=\frac{2\times N_{P}}{3\times N_{RP}}. \end{array}$$

With fixed $N_{P}$, L is shorter for a higher link% system, and 1/L can also be used to quantify the cross-linking density. Since the main objective was to generate the cross-linking structure for the polymer host, the starting polymer length was set to L and only the cross-linking process was considered. The cross-linking reaction was terminated when the yield of the new bonds was above 95%. The detailed system compositions for systems with different cross-linking densities are listed in Table 2.

The initial configurations of solutes in polymers before the cross-linking process were randomly placed into a cubic cell with the initial density of 0.9 $\sigma^{-3}$ according to the composition listed in Table 2. Each system was first energy minimized through the steepest descent minimization algorithm, followed by an isothermal-isobaric (NPT) simulation at the temperature T = 1.0 $\epsilon /k_{B}$ and the pressure P = 0 $\epsilon /\sigma^{3}$ for 25,000 τ where τ denotes the LJ reduced unit of time. The system was then cross-linked under the same NPT condition for a minimal 50,000 τ until the linking yield reached above 95%. The system was then equilibrated for 25,000 τ at various temperatures ranging from 0.3 to 0.975 with intervals of 0.025 $\epsilon /k_{B}$. The last 20,000 τ simulation was used for data analyses.

All simulations were carried out using the Large-scale Atomic/Molecular Massively Parallel Simulator (LAMMPS) software with an integration time step of 0.005 τ [34]. Temperature and pressure were controlled using the Nos$\overset{´}{e}$-Hoover thermostat and barostat with the damping parameters of 0.5 τ and 5 τ, respectively [35,36,37]. System configurations were saved every 25 τ for further analyses. Simulations were visualized with the visual molecular dynamics (VMD) software [38]. All the analyses described in the subsequent sections were performed using in-house analysis scripts.

### 2.2. Free Volume Theory for Diffusion

A common interpretation of the VTF equation is based on the free volume theory first proposed by Cohen and Turnbull [17,18]:

(10) $$\begin{array}{c} {D=D_{F}\;e}^{-\gamma V^{*}/V_{f}}. \end{array}$$

Here, $V^{*}$ denotes the critical cavity for solute diffusion, $V_{f}$ is the mean cavity size, and γ is a correction factor between 0.5 and 1 accounting for cavity overlaps. Moreover, the pre-exponential factor D${}_{F}$ is related to solute velocity, molecular diameters, and a geometric correction. Mean volume $V_{f}$ is assumed to expand linearly with temperature:

(11) $$\begin{array}{c} V_{f}=\alpha V_{m}\left(T-T_{0}^{\prime }\right), \end{array}$$

where α is the thermal expansion coefficient, $V_{m}$ is the mean molecular volume, and $T_{0}^{\prime }$ denotes the extrapolated temperature at which the solute free volume presumably disappears. Comparing Equation (3) with the combination of Equations (10) and (11) and assuming $T_{0}^{\prime }$ = T${}_{0}$, we can obtain the expression of the following:

(12) $$\begin{array}{c} E_{a}/R=\gamma V^{*}/\alpha V_{m}, \end{array}$$

which directly correlates the free volume with the pseudo-activation energy of VFT diffusion.

**Figure 2:**
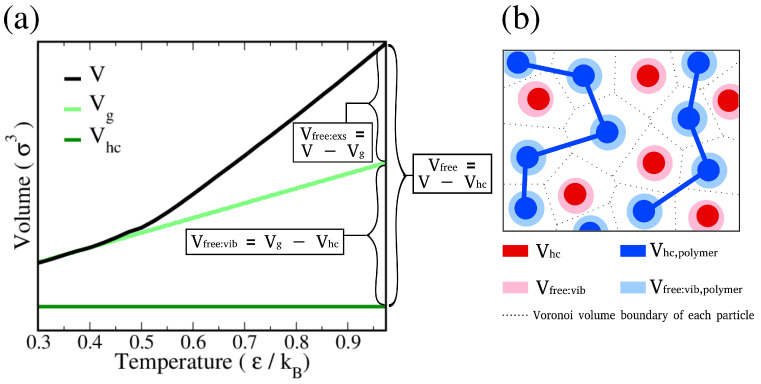
(**a**) A representative solute volume thermogram (V-T plot), labeled with different solute volume definitions. (**b**) Illustrations of different definitions of free volumes, where the volumes for solutes and polymers are marked with red-ish and blue-ish colors, respectively.

Free volume theory has been first proposed to interpret the behavior of polymer glass transition. In analogy to the interpretation of polymer free volume by White and Lipson [39], we defined the free volume of solute $V_{free}$ in the polymer hosts as follows:

(13) $$\begin{array}{c} V_{free}\;=V-V_{hc}=V_{free:vib}+V_{free:exs}, \end{array}$$

where *V* is the total volume occupied by a solute evaluated using the Voronoi analysis here [40]. Note that *V* is temperature-dependent, where the thermal expansion coefficients above and below $T_{g}$ are different, as illustrated in Figure 2. $V_{hc}$ denotes the vibration-free, hard-core volume of solute and is independent of temperature. Here, we evaluated $V_{hc}$ by extrapolating the *V*-T curve linearly to T = 0. The solute free volume thus can be calculated as the difference between *V* and $V_{hc}$ which represents the maximum free volume [39]. Note that, compared to $V_{hc}$, the local vibration of molecules can lead to a slightly larger effective volume as in glassy state, namely $V_{g}$ [41,42,43]. Additionally, molecules are nearly frozen and only vibrate locally below $T_{g}$. Hence, we assumed *V* of solute to be equal to $V_{g}$ below $T_{g}$. This allowed the estimation of $V_{g}$ at any temperature through the linear extrapolation of the *V*-T curve below $T_{g}$. As shown in Equation (13), the maximum solute free volume $V_{free}$ can be further divided into the vibrational free volume $V_{free:vib}$ and the excess free volume $V_{free:exs}$, where $V_{free:vib}$ was evaluated by the difference between $V_{g}$ and $V_{hc}$, and $V_{free:exs}$ was defined as the difference between *V* and $V_{g}$ [39]. Figure 2 demonstrates the definitions of different free volumes and summarizes the relations among them.

Based on the free volume theory of diffusion, the transport of solute occurs only in the space exclusive to the polymer chains. Therefore, the total free volume available for solute motion, as demonstrated in Figure 2b, should account for both the volume of solute and the excess free volume of polymers. Here, we estimated the total volume available for each solute molecule $V^{D}$ and the volume available for diffusion $V_{free}^{D}$ as:

(14) $$\begin{array}{c} V^{D}=\frac{V_{free:exs,polymer}\times \left(N_{P}+N_{RP}\right)+V\times N_{C}}{N_{C}},\;\mathrm{and} \end{array}$$

(15) $$\begin{array}{c} V_{free}^{D}=V^{D}-V_{hc}, \end{array}$$

where $V_{free}^{D}$ excludes the hard-cord volume of each solute from $V^{D}$. Using Equation (11) and assuming $V_{free}^{D}=V_{f}$, the $\alpha V_{m}$ value was then estimated from the slope of the $V_{free}^{D}$-T plot. The correlation between $E_{a}/R$ and $V_{free}^{D}$ was then validated using Equation (12).

## 3. Results and Discussions

### 3.1. Solute Diffusion in Cross-Linked Polymers

**Figure 3:**
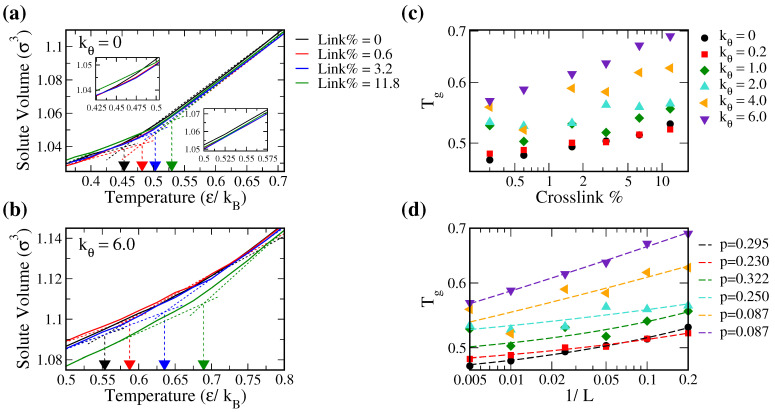
Representative solute volume thermograms (*V* versus T) for (**a**) the soft polymer ($k_{\theta }=0$) and (**b**) the stiff polymer ($k_{\theta }=6.0$) with Link% of 0, 0.6, 3.2, and 11.8%, where the insets in (**a**) emphasize the regions both above and below $T_{g}$. The glass transition temperature $T_{g}$ determined via intersecting the linear fitted lines at high and low temperature regions for each system is marked by the triangle on the x-axis. $T_{g}$ plotted against (**c**) Link% and (**d**) inverse of linker length 1/L for all tested polymer rigidities. Values of the power coefficient p in Equation (16) obtained from fitting $T_{g}$-1/L curves are also provided.

As discussed earlier, the solute diffusion modes are different below and above $T_{g}$. We first determined $T_{g}$ from the molecular volume *V*-temperature T plots. Figure 3a,b display the representative *V*-T plots for the softest ($k_{\theta }$ = 0.0) and the most rigid ($k_{\theta }$ = 6.0) polymers, respectively. Since we were interested in the solute diffusion, specifically the type C solute as discussed in Methods, we evaluated *V* using the Voronoi volume of solute C. The solute $T_{g}$ determined by the intersection between the linear fitted lines at high and low T regions were also labeled in Figure 3a,b.

For the softest chain ($k_{\theta }$ = 0) systems, at T below $T_{g}$, *V* is less affected by Link%, except for Link% = 11.8 where *V* increases. This suggested that the free volume of solute is less affected by cross-linking in soft polymers in glassy state. Yet, with a high cross-linking density, the linker length L is shorter to provide a more rigid and less compressible polymer framework, leading to increased solute volume. When above $T_{g}$, *V* is reduced if polymers start cross-linking but is less affected by further varying Link%. In the rubbery phase above $T_{g}$, the motion of solute is associated with polymer mobility. If cross-linked, the polymer segments are restrained with less effective occupied volumes, leading to a reduced *V* of solute. In contrast, in rigid polymer systems, *V* decreases as Link% increases both below and above $T_{g}$. The molecular packing within rigid polymers is less dense both below and above $T_{g}$. Thus, introducing cross-linking restraints can enforce smaller chain separations at all temperatures, resulting in reduced *V* of solute.

$T_{g}$ values with various Link% for polymers of different rigidities are presented in Figure 3c. Clearly, $T_{g}$ increases with increasing Link%. This is because the chain segmental motions are restrained by cross-linking and more thermal energy is required to transition into the rubbery state in which the chain motions are coupled with the solute transport. Conventionally, $T_{g}$ of cross-linked polymers is related to the linker length as $T_{g}=T_{g0}+A/L$, where $T_{g0}$ is the $T_{g}$ of uncrosslinked system and A is the empirical constant. Note, however, the $T_{g}$ evaluated in this work was based on *V* of solute and not for pure polymer melts. Therefore, as illustrated in Figure 3d, the obtained $T_{g}$s were proportional to 1/L${}^{n}$ with n < 1:

(16) $$\begin{array}{c} T_{g}=\frac{c_{1}}{L^{{}^{p}}}+c_{2}. \end{array}$$

Here, c${}_{2}$ represents the solute $T_{g}$ of uncrosslinked systems. Power p is affected by polymer rigidity: p ≈ 0.09 for stiff polymers ($k_{\theta }=4.0,6.0$) and p ≈ 0.2∼0.3 for soft polymers ($k_{\theta }\leq 2.0$). This suggests that cross-linking may affect differently on solute transport within polymers with various rigidities.

**Figure 4:**
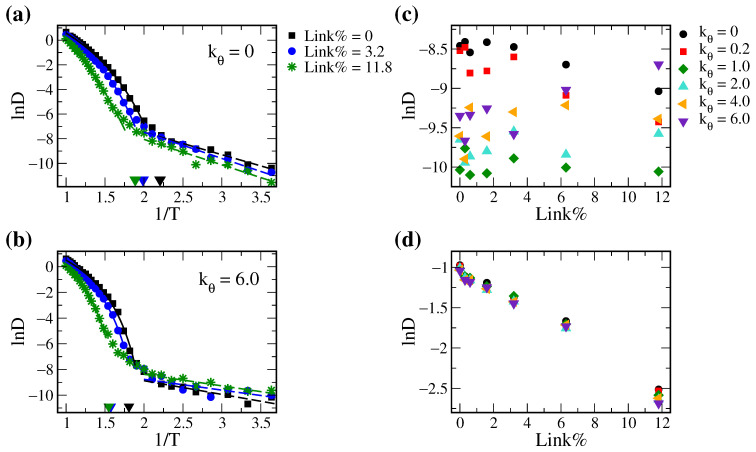
Representative Arrhenius plots (lnD versus 1/T) for (**a**) the soft polymer ($k_{\theta }=0$) and (**b**) the stiff polymer ($k_{\theta }=6.0$) with Link% of 0, 3.2, and 11.8%, where the glass transition temperature $T_{g}$ for each system is marked by the triangle on the x-axis. Natural logarithm of the solute diffusion coefficient D measured at (**c**) T = 0.4 and (**d**) T = 0.75 versus Link% for all polymeric systems with various rigidities.

We evaluated the diffusivity of solute C in the polymer matrices with different Link% at various temperatures ranging from 0.3 to 0.975. Figure 4a,b show the representative Arrhenius plots for the softest and the most rigid polymers with three different Link%, respectively. Below $T_{g}$, the solute diffusion follows the Arrhenius behaviors with the linear lnD to 1/T relation for all systems. Above $T_{g}$, in contrast, solute in all tested systems diffuse in the VTF mode with non-linear lnD-1/T curves. This suggests that polymer segmental motions play an important role in solute diffusion despite polymer rigidity or cross-linking density.

Figure 4c,d summarize the calculated solute diffusion coefficients D at T = 0.4 < $T_{g}$ and T = 0.75 > $T_{g}$, respectively, for all tested polymer systems. Below $T_{g}$, D decreases with increasing Link% for soft polymeric systems, i.e., $k_{\theta }=0.0,0.2$. Yet, such a trend starts to inverse as the chain rigidity increases. For polymers with $k_{\theta }=6.0$, D increases as Link% increases at T < $T_{g}$. In contrast, the calculated D values above $T_{g}$ appear to decrease with increasing Link% for all tested polymer rigidity. Note, however, D for polymers with moderate rigidities ($k_{\theta }=1.0,2.0$) are slightly higher than other systems with the moderate cross-linking density (Link% = 0.3∼3.2). This suggested that it is possible to modulate both polymer rigidity and Link% and maximize the solute diffusion within polymer matrix.

### 3.2. Beyond Compensation Effect in the VTF Model

**Figure 5:**
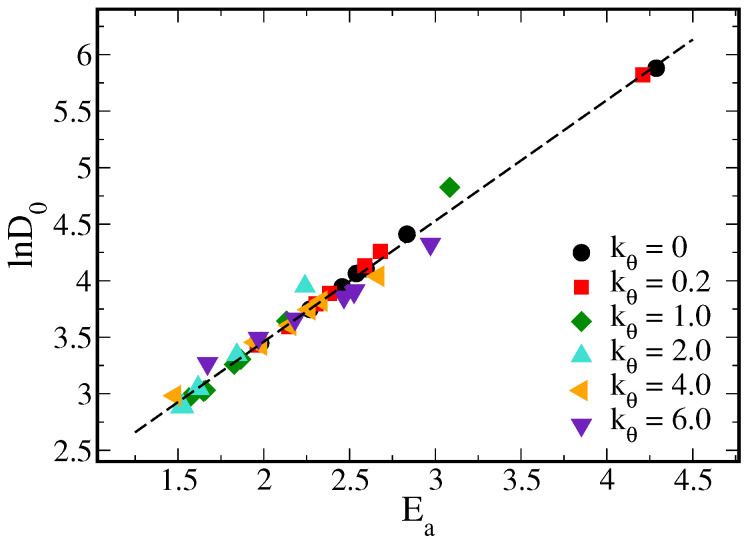
Natural logarithm of the VTF pre-exponential factor D${}_{0}$ versus the pseudo-activation energy $E_{a}$ for all tested polymer systems in rubbery states.

In most polymer electrolyte systems, it is known that polymers in rubbery state have higher ion conductivity. Therefore, here, we focused on studying the effects of polymer rigidity and cross-linking on solute diffusion above $T_{g}$. We fitted the Arrhenius plots for all tested systems above their corresponding $T_{g}$ with the VTF equation (Equation (3)) . The three parameters, i.e., D${}_{0}$, $E_{a}$, and $T_{0}$, were determined by linear fitting the lnD${}_{0}$-1/(T−T${}_{0}$) curve with the maximum coefficient of determination $R^{2}$. Figure 5 plots the resulting ln(D${}_{0}$) versus $E_{a}$ from all tested systems. The results show a clear compensation relation following Equation (2), with the coefficient m=1.069 and n=1.324. This result indicates that the polymer rigidity and cross-linked network have minimal effects on D${}_{0}$-$E_{a}$ compensation. Combining our result with the study by Diederichsen et al. on the compensation relations for various polymer electrolyte systems [19], other factors such as the ion-polymer affinity and microstructures within polymer blends should be more dominant factors on the D${}_{0}$-$E_{a}$ compensation effect.

**Figure 6:**
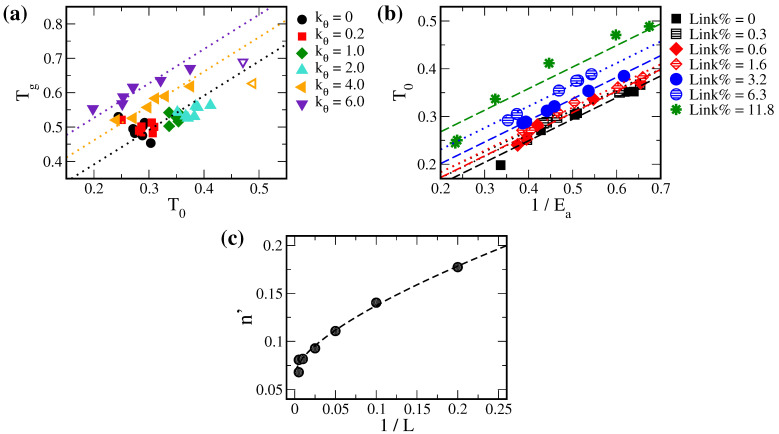
(**a**) The glass transition temperature $T_{g}$ versus the Vogel temperature T${}_{0}$ for all tested polymer solutions. Fitting the curves with the relation $T_{g}$ = T${}_{0}$ + ΔT, polymers with $k_{\theta }$ = 0, 0.2, 1.0, and 2.0 were fitted with one ΔT. Polymers with $k_{\theta }$ = 4.0 and 6.0 were fitted separately to obtain two ΔT values, where the systems with the highest Link% (hollowed symbols) showed much deviations and were excluded from fitting. (**b**) T${}_{0}$ versus 1/$E_{a}$ for all tested systems. The linear function (Equation (18)) was fitted to systems with the same Link%. (**c**) The coefficient n${}^{\prime }$ in Equation (18) versus the inverse linker length 1/L. The data were then fitted with the function $\frac{c_{3}}{L^{{}^{p^{\prime }}}}+c_{4}$ as in Equation (18).

Experimentally, the Vogel temperature T${}_{0}$ in the VTF equation is typically taken as follows:

(17) $$\begin{array}{c} T_{0}=T_{g}-\Delta T=\frac{c_{1}}{L^{{}^{p}}}+c_{2}-\Delta T, \end{array}$$

where ΔT is an empirical value of 50 K [15]. Figure 6a plots the $T_{g}$ with the corresponding T${}_{0}$ for all systems tested in this work. Indeed, we found a distinct $T_{g}=T_{0}+\Delta$T relation where ΔT is less affected by polymer cross-linking. Therefore, the empirical ΔT constant of 50 K should be valid for most polymer systems. However, ΔT increases for highly rigid polymer systems, i.e., $k_{\theta }=4.0,6.0$. This suggests that high polymer stiffness can have a more dominant effect on T${}_{0}$ in the VTF equation. Combined with the empirical relation of Equation (16) from Figure 3c,d, we further related T${}_{0}$ with the cross-linking density 1/L as the second equality of Equation (17).

According to the *V* thermograms in Figure 3, solute volume *V* is affected by Link%. Since the solute free volume is related to $1/E_{a}$ based on the free volume theory (Equation (12)), there may be a correlation between T${}_{0}$ and $E_{a}$. Indeed, as illustrated in Figure 6b, we identified a second linear correlation in VTF parameters other than Equation (2) between T${}_{0}$ and 1/$E_{a}$:

(18) $$\begin{array}{c} T_{0}=\frac{m^{\prime }}{E_{a}}+n^{\prime }=\frac{m^{\prime }}{E_{a}}+\frac{c_{3}}{L^{{}^{p^{\prime }}}}+c_{4}. \end{array}$$

Here, n${}^{\prime }$ denotes the Vogel temperature for systems with an infinite activation energy of diffusion when the mean solute volume disappears, according to Equation (12). Further analyses showed that slope m${}^{\prime }$ remained constant for all systems yet the intercept n${}^{\prime }$ was dependent on Link%. Moreover, n’ is proportional to $1/L^{{}^{p^{\prime }}}$, as illustrated in Figure 6c. Thus, other than the conventional compensation between D${}_{0}$ and $E_{a}$, Equation (18) provides a second correlation between $E_{a}$ and $T_{0}$ in the VTF equation.

### 3.3. Solute Diffusion and Free Volume

**Figure 7:**
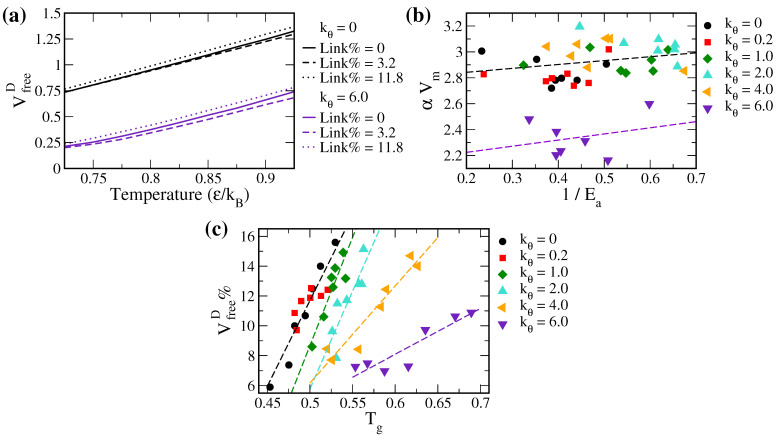
(**a**) Representative $V_{free}^{D}$ thermograms for the soft ($k_{\theta }$ = 0) and the stiff ($k_{\theta }$ = 6.0) polymers with Link% of 0, 3.2, and 11.8 in the T range of 0.725 to 0.925. (**b**) $\alpha V_{m}$ versus $1/E_{a}$ for all tested polymer systems. The purple dashed line denotes the linear fit for the polymer systems of $k_{\theta }$ = 6.0; while the black dashed line is the linear fit for all other polymer systems. (**c**) $V_{free}^{D}\%$ versus T${}_{g}$ for all tested polymer systems. Data were fitted to a linear function based on the polymer rigidity. Data of $k_{\theta }$ = 0 and 0.2 systems were joined and fitted together.

To further analyze the free volume theory for diffusion, we used the thermal expansion of $V_{free}^{D}$ to evaluate the value of $\alpha V_{m}$, i.e., the product of the thermal expansion coefficient and the mean molecular volume. As illustrated in Figure 7a, we estimated $\alpha V_{m}$ via fitting $V_{free}^{D}$ thermograms in the temperature range of 0.725 to 0.925. According to Equation (12), $\alpha V_{m}$ should be inversely proportional to the pseudo activation energy of diffusion $1/E_{a}$. However, as shown in Figure 7b, the resulting $\alpha V_{m}$ values are not well correlated with $1/E_{a}$. Note that $E_{a}$ is also affected by γ, the correction factor for the free cavity overlaps. The poor correlation between $\alpha V_{m}$ and $1/E_{a}$ thus suggests that γ can also be temperature-dependent, which will be investigated in our future studies.

According to the study by White and Lipson [39], the glass transition temperature $T_{g}$ of a polymer melt is related to the free volume of the polymer segment at $T_{g}$. They found a near-linear correlation between the free volume ratio $V_{free}\%=\left(V_{free}/V\right)\times 100\%$ and $T_{g}$. Such a linear relationship represents the “minimum free volume percentage”, which is the free volume threshold, for polymers in a melt state. In analogy to the free volume theory for solute diffusion, the “free volume threshold” can be interpreted as the transition between the Arrhenius and the VTF type diffusive behaviors. Here, we calculated the total volume and free volume for solute diffusion using Equations (14) and (15), respectively, and evaluated the free volume ratio $V_{free}^{D}\%=\left(V_{free}^{D}/V^{D}\right)\times 100\%$. As shown in Figure 7c, $V_{free}^{D}\%$ is linearly related to $T_{g}$ as follows:

(19) $$\begin{array}{c} V_{free}^{D}\%=m^{\prime \prime }T_{g}+n^{\prime \prime }, \end{array}$$

where the slope m${}^{\prime \prime }$ and the intercept n${}^{\prime \prime }$ are both dependent on polymer rigidity. This suggests that the boundary between two different solute transport mechanisms can be modulated by polymer stiffness. Note that the rigidity dependency is different from the Link% dependency in Figure 6, which may be due to the poor correlation between $\alpha V_{m}$ and $1/E_{a}$ discussed above. Assuming $V_{free}^{D}=V_{f}\propto 1/E_{a}$, the above linear correlation thus provides a rationale for the linear relation in Equation (18).

### 3.4. Optimization of Solute Diffusion in Cross-Linked Polymers

The crossed relations among D${}_{0}$, T${}_{0}$, and $E_{a}$ allow us to re-formulate the VTF equation for optimizing the solute diffusivity. Table 3 summarizes the correlations among the VTF parameters and dependencies of the corresponding coefficients on polymer rigidity, where the values for all parameters are listed in Tables S1 and S2 of Supplementary Material. Combining the correlation between T${}_{0}$ and 1/L in Equation (17) and the one between T${}_{0}$ and $E_{a}$ in Equation (18), we derived the relation between $E_{a}$ and the cross-linking density 1/L as follows:

(20) $$\begin{array}{c} E_{a}=\frac{m^{\prime }}{c_{1}/L^{{}^{p}}-c_{3}/L^{{}^{p^{\prime }}}+c_{2}-c_{4}-\Delta T}. \end{array}$$

**Figure 8:**
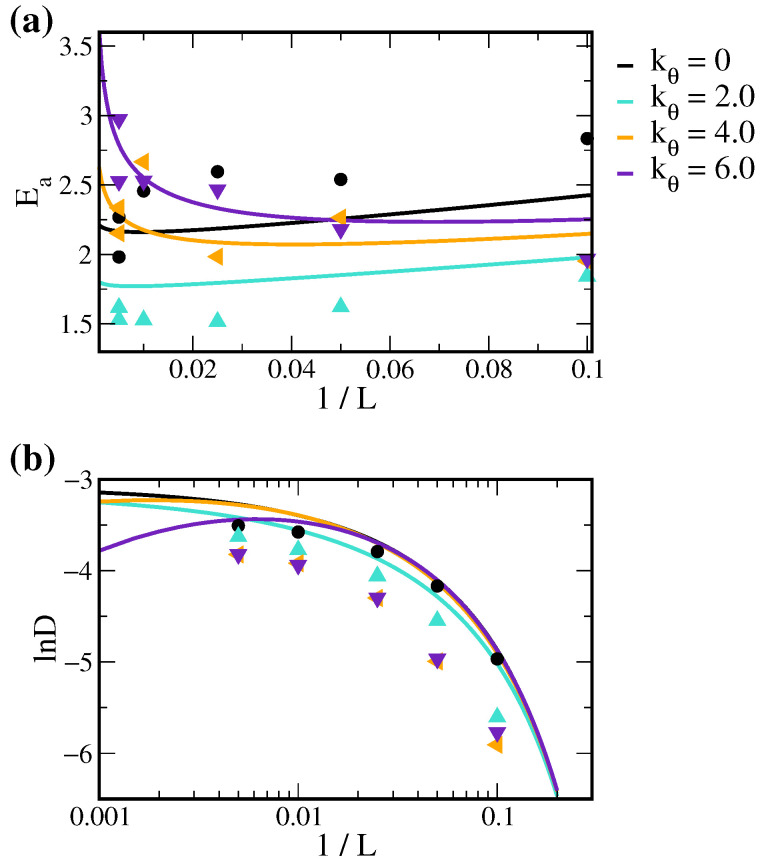
The theoretical predictions of (**a**) $E_{a}$ and (**b**) lnD as functions of 1/L for different $k_{\theta }$ according to Equation (20) and Equation (21), respectively. The points denote the data obtained from MD simulations in this work.

Figure 8a illustrates the $E_{a}$-1/L correlations for various $k_{\theta }$ values using the empirical coefficients obtained in this work. The predicted $E_{a}$ shows more distinct variations with respect to 1/L changes for rigid polymers. Additionally, as cross-linking density increases in soft polymers, $E_{a}$ first slightly decreases and then gradually increases. For rigid polymer systems, $E_{a}$ first drastically decreases and then slightly increases. These results demonstrate how polymer rigidity alters the activation energy of diffusion.

Combining Equations (2) and (18), we reformulated the VTF equation of Equation (3) as follows:

(21) $$\begin{array}{c} \ln\left(D\right)={\mathrm{mE}}_{a}+n-\frac{E_{a}}{R(T-c_{1}/L^{{}^{p}}-c_{2}+\Delta T)}. \end{array}$$

Further substituting Equation (20) into the above equation leads to the direct relation between the diffusion coefficient D and cross-linking density 1/L. As shown in Figure 8b, solute diffusivity monotonically decreases with increased cross-linking density for soft polymers. In contrast, solute diffusivity exhibits a maximum value at moderate cross-linking density for rigid polymer systems.

According to VTF equation Equation (3), increasing $E_{a}$ or $T_{0}$ leads to reduced D. From Equation (17), a higher cross-linking density corresponds to an increased T${}_{0}$. However, from the $E_{a}$-1/L relations in Figure 8a, a rapid decrease in $E_{a}$ while the cross-linking starts in rigid polymer systems. This suggests that, for polymers with higher stiffness, introducing a low degree of cross-linking can increase the solute diffusions in polymer melts. The competing effects between $E_{a}$ and T${}_{0}$ also lead to an optimal cross-linking density for solute diffusion. In contrast, cross-linking soft polymers have a negligible reduction on $E_{a}$ at low 1/L and thus only reduce the solute mobility as Link% increases. Note that Equations (20) and (21) only moderately fit with simulation results as shown in Figure 8, where the errors can be due to the accumulation of numerical errors from parameter fitting and the complex dependency on cross-linking density for parameters in the free volume theory of solute diffusion within polymers as discussed in Section 3.3.

## 4. Conclusions

In this study, we utilized a series of coarse-grained molecular dynamics to systematically examine the effects of cross-linking and polymer rigidity on solute diffusivity within host polymers. The simulation results showed that the glass transition temperature of solute $T_{g}$ is related to the cross-link density 1/L as Equation (16), where the coefficients are dependent on polymer stiffness. Kinetic analyses illustrated that when above >$T_{g}$, solute diffusion follows typical VTF behavior as shown in Equation (3). Via fitting MD data with the VTF equation, the resulting pseudo activation energy $E_{a}$ and the pre-exponential factor D${}_{0}$ exhibited a conventional compensation correlation of Equation (2). Consistent with experimental results, the obtained Vogel temperature coefficient T${}_{0}$ was lower than $T_{g}$ by a constant ΔT. Yet, ΔT was found to be dependent on polymer rigidity. Furthermore, we identified an additional correlation among T${}_{0}$, $E_{a}$, and the cross-link density 1/L as described in Equation (17). These results allowed us to derive an empirical relation between $E_{a}$ and the cross-link density (Equation (20)) and to re-formulate the VTF equation into the correlation between the solute diffusivity and the polymer cross-linking density (Equation (21)). With such newly derived correlation, we found that increasing the cross-link density in soft polymers monotonically reduces solute diffusivity. In contrast, there exhibits an optimal cross-link density maximizing the solute transport properties in rigid polymers. Future work includes mapping the correlation among the practical polymer rigidity characters, such as persistent length, and the aforementioned coefficients and studying the underlying physics. The effects of polymer rigidity and cross-linking on the cavity overlap parameter γ in the free volume theory of diffusion will also be further examined. The combined results can provide valuable guidance for the optimization of polymeric materials for various applications, including novel polymer electrolytes for energy devices.

## Figures and Tables

**Table 1:** Non-bonded interaction parameters.

Type *i*-*j*	$\varepsilon_{\mathrm{ij}}\left(\epsilon \right)$	$\sigma_{\mathrm{ij}}\left(\sigma \right)$	Potential Form
P-P	1.0	1.0	LJ
C-C	1.0	1.0	WCA
A-A	1.0	1.0	WCA
P-C	2.0	1.0	LJ
P-A	1.0	1.0	LJ
C-A	2.0	1.0	LJ

**Table 2:** Compositions for polymer solutions with various Link%.

Link%	Polymer Chain Length (L) × Chain Number	Reactive Polymer Chain Length (L+2) × Chain Number	$N_{P}$	$N_{\mathrm{RP}}$	$N_{C}$	$N_{A}$
0	200×150	202×0	30,000	0	1875	1875
0.3	200×100	202×50	30,000	100	1875	1875
0.6	100×200	102×100	30,000	200	1875	1875
1.6	40×50	42×250	30,000	500	1875	1875
3.2	20×1000	22×500	30,000	1000	1875	1875
6.3	10×2000	12×1000	30,000	2000	1875	1875
11.8	5×4000	7×2000	30,000	4000	1875	1875

**Table 3:** The correlations among the VTF parameters where the coefficients depending on polymer rigidity are also listed.

Correlation	Polymer Rigidity Dependent Coefficients
$\ln\left(D_{0}\right)={\mathrm{mE}}_{a}+n$ (Equation (2))	−
$T_{0}=c_{1}/L^{{}^{p}}+c_{2}-$ΔT (Equation (17))	$c_{1}$, $c_{2}$, p, ΔT
$T_{0}=m^{\prime }/E_{a}+c_{3}/L^{{}^{p^{\prime }}}+c_{4}$ (Equation (18))	−

## Data Availability

The simulation data supporting the findings of this study are available from the corresponding author upon reasonable request.

## Supplementary Materials

The following supporting information can be downloaded at: https://www.mdpi.com/article/10.3390/polym14102061/s1, Table S1: Rigidity independent correlations and coefficients; Table S2: Rigidity dependent correlations and coefficients.
