# Supplementary material for: Solute Diffusivity and Local Free Volume in Cross-Linked Polymer Network: Implication of Optimizing the Conductivity of Polymer Electrolyte
Source: Polymers (Basel). 2022 May 18;14(10):2061. doi: 10.3390/polym14102061 (PMC9145971; doi:10.3390/polym14102061)
Supplement: Supplementary file 1 [file polymers-14-02061-s001.zip › SI_Crosslink.pdf]

Supplementary Materials

for

Solute Diffusivity and Local Free Volume in Cross-Linked Polymer Network: Implication of Optimizing the Conductivity of Polymer Electrolyte

Yi-Chen Tsai [1,2,†] and Chi-cheng Chiu [1,2,3,†,*]

1 Department of Chemical Engineering, National Cheng Kung University, Tainan 70101, Taiwan; a4775324@gmail.com (Y.-C. T.)

2 Hierarchical Green-Energy Materials (Hi-GEM) Research Center, National Cheng Kung University, Tainan 70101, Taiwan

3 Fire Protection of Safety Research Center, National Cheng Kung University, Tainan 711, Taiwan

* Correspondence: ccchiu2@mail.ncku.edu.tw

† These authors contributed equally to this work.

Table S1. Rigidity independent correlations and coefficients

Correlations	Fitting coefficients
$lnD_0 = mE_a + n$ (Eq. 2)	$m = 1.069,\ n = 1.324$
$T_0 = m'/E_a + c_3/L^{p'} + c_4$ (Eq. 18)	$m' = 0.453,\ c_3 = 0.327,\ p' = 0.677,$ $c_4 = 0.0685$

Table S2. Rigidity dependent correlations and coefficients

Correlation: $T_0 = c_1/L^p + c_2 - \Delta T$ (Eq. 17 in main text)

$\boldsymbol{k_\theta}$	$\boldsymbol{c_1}$	$\boldsymbol{c_2}$	$\boldsymbol{p}$	$\boldsymbol{\Delta T}$
0	0.126	0.451	0.295	0.191
0.2	0.088	0.460	0.230	0.191
1.0	0.125	0.479	0.322	0.191
2.0	0.112	0.494	0.250	0.191
4.0	0.384	0.295	0.087	0.261
6.0	0.524	0.236	0.087	0.327
